# State‐of‐Charge Distribution of Single‐Crystalline NMC532 Cathodes in Lithium‐Ion Batteries: A Critical Look at the Mesoscale

**DOI:** 10.1002/cssc.202201169

**Published:** 2022-10-01

**Authors:** Till‐Niklas Kröger, Mathis Jan Wölke, Patrick Harte, Thomas Beuse, Martin Winter, Sascha Nowak, Simon Wiemers‐Meyer

**Affiliations:** ^1^ MEET Battery Research Center University of Münster Corrensstraße 46 48149 Münster Germany; ^2^ Helmholtz-Institute Münster IEK-12 FZ Jülich Corrensstraße 46 48149 Münster Germany

**Keywords:** electrochemistry, energy storage, lithium-ion batteries, particle size, state of charge

## Abstract

The electrochemical response of layered lithium transition metal oxides LiMO_2_ [M=Ni, Mn, Co; e. g., Li(Ni_0.5_Mn_0.3_Co_0.2_)O_2_ (NMC532)] with single‐crystalline architecture to slow and fast charging protocols and the implication of incomplete and heterogeneous redox reactions on the active material utilization during cycling were the subject of this work. The role of the active material size and the influence of the local microstructural and chemical ramifications in the composite electrode on the evolution of heterogeneous state of charge (SOC) distribution were deciphered. For this, classification‐single‐particle inductively coupled plasma optical emission spectroscopy (CL‐SP‐ICP‐OES) was comprehensively supplemented by various post mortem analytical techniques. The presented results question the impact of surface‐dependent failure mechanisms of single crystals for the evolution of SOC heterogeneity and identify the deficient structural flexibility of the composite electrode framework as the main driver for the observed non‐uniform active material utilization.

## Graphical abstract

**State of charge**: The particle size‐ and rate‐dependent evolution of persistent mesoscale state‐of‐charge heterogeneity is revealed upon different cycling protocols for NMC532 with single‐crystalline architecture. The structural fatigue of the composite matrix is concluded to be the main driver for the observed non‐uniform active material utilization.

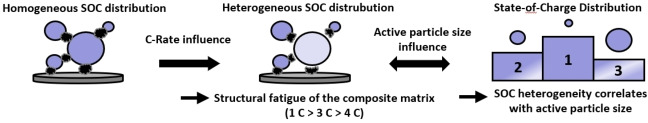

## Introduction

The strategy of developing micron‐sized cathode active material (CAM) particles with single‐crystalline architecture is contemplated to overcome the limitations of the prevailing polycrystalline microstructure.[^1^, ^2^] The deteriorating electrochemical performance of polycrystalline Ni/Mn/Co (NMC)‐based cathode materials is often attributed to the structural decay of the mesoscale architecture upon cycling.[^1^, ^3^] The evolution of micro‐cracks is accompanied by several implications (e. g., cathode|electrolyte interfacial degradation, contact loss) leading to dysfunctional redox reactions of the CAM.[^1^, ^3^, ^4^, ^5^, ^6^, ^7^, ^8^, ^9^, ^10^] The monolithic architecture of the single crystals strengthens the microstructure, resulting in higher robustness to the anisotropic stress development during (de)lithiation and thus improved cycling stability.[^1^, ^2^, ^6^, ^11^, ^12^] Although single‐crystalline NMCs are characterized by an outstanding durability upon long‐term cycling and are lauded in the literature as a replacement of the polycrystalline microstructure, the rate capability properties of this structure type are questioned.^[13]^ Hierarchically structured polycrystals are composed of multiple primary particles with short transport ways intertwined by a network of grain boundaries enabling fast three‐dimensional charge carrier transport.^[14]^ In contrast, the kinetic limitation to the rate performance of single crystals due to the long lithium transport paths along the layer planes in the active particles is a major drawback of this microstructure for fast cycling.[^6^, ^13^, ^14^, ^15^] Among physico‐chemical properties such as the lithium diffusivity and electron conductivity in the active material, the local electrochemistry is further defined by the complexity of the composite electrode microenvironment.[^4^, ^16^, ^17^, ^18^, ^19^, ^20^, ^21^] Lithium transition metal oxides as performance materials do not fail homogeneously; in fact, nano‐ and microscale heterogeneities interact at the mesoscale to cause failure at device level.[^22^, ^23^, ^24^] The structural and chemical heterogeneity of the CAM particles and the composite electrode, whether inherently present or evolved during aging, significantly affects the mesoscale lithium transport pathways and can ultimately lead to state‐of‐charge (SOC) heterogeneity.[^10^, ^25^, ^26^] There is a plethora of reasons for the evolution of mesoscale SOC heterogeneity including the formation of inactive surface phases [e. g., surface reconstruction, erratic cathode electrolyte interphase (CEI) growth], deficient electrical contact, or the persistent confinement of lithium reservoirs by mechanical disintegration.[^5^, ^9^, ^27^, ^28^, ^29^, ^30^, ^31^, ^32^, ^33^, ^34^] In this study, the SOC distribution of CAM particles with single‐crystalline architecture after cycling at different rates is investigated by means of classification‐single‐particle inductively coupled plasma optical emission spectroscopy (CL‐SP‐ICP‐OES).^[35]^ The role of the particle size for the electrochemical reactions is of paramount importance as smaller sizes lead to better mechanical integrity during charge/discharge cycling and improved transport kinetics, still noting the increased influence of surface degradation due to deleterious cathode|electrolyte interfacial reactions.[^6^, ^36^, ^37^, ^38^, ^39^] The systematic investigation of the particle size influence on the mesoscale SOC distribution is enabled by size fractionation via low‐pressure cascade impaction. Furthermore, time of‐flight secondary ion mass spectrometry (ToF‐SIMS), scanning electron microscopy (SEM), and energy‐dispersive X‐ray spectroscopy (EDX) are utilized to holistically investigate the local microstructure and chemical environment and their implications on the mesoscale SOC distribution.

## Results and Discussion

### Investigation of the mesoscale SOC heterogeneity between different particles upon delithiation

**Figure 1:**
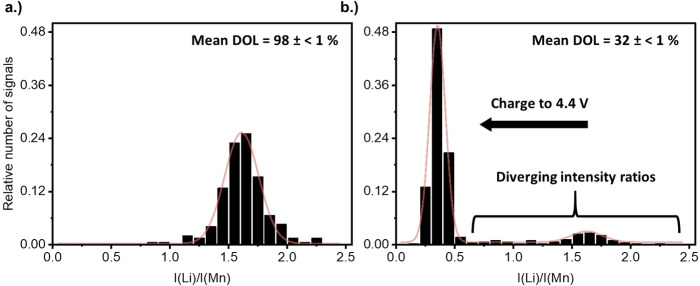
Relative histograms of the NMC532 particle intensity ratios before size fractionation by cascade impaction of (a) pristine and (b) electrochemically delithiated NMC532 (SOC of 80±1 %) in NMC532‖graphite cells with the mean DOL as obtained by CL‐SP‐ICP‐OES and after acidic microwave digestion with ICP‐OES in solution mode, respectively. The red line indicates the Gaussian fit.

The mesoscale SOC distribution of the NMC532 is investigated using the intensity ratio of Li and Mn [*I*(Li)/*I*(Mn)], which is determined by CL‐SP‐ICP‐OES and calibrated with a matrix‐matched external calibration to obtain the degree of lithiation (DOL) of individual CAM particles. For the matrix‐matched external calibration, differently lithiated NMC532 is provided by electrochemical delithiation (see Figure S1). The mean intensity ratios decrease linearly with lower mean DOLs, which validates the applicability of the calibration approach. In Figure 1, the relative histograms of the intensity ratios of pristine and electrochemically delithiated NMC532 particles before size fractionation by cascade impaction are presented. The intensity ratios in the relative histogram of the pristine NMC532 particles are characterized by a monomodal Gaussian distribution. However, after electrochemical delithiation at a slow rate and extensive time for relaxation, a bimodal distribution with lower and higher intensity ratios is obtained. The assumption of homogeneously occurring redox reactions in the CAM would imply uniform delithiation. Therefore, all the measured intensity ratios of the CAM particles are expected to follow a monomodal Gaussian distribution.

**Figure 2:**
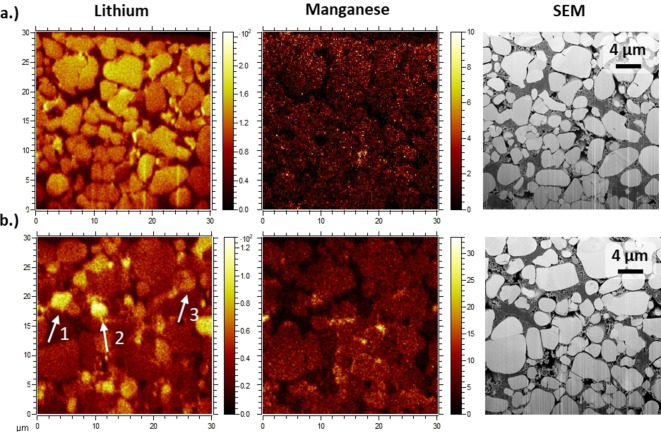
ToF‐SIMS mappings for a FIB‐prepared cross‐section of (a) pristine and (b) electrochemically delithiated NMC532 electrode (SOC of 80±1 %) from NMC532‖graphite cells. SEM and secondary ion images for Li species (^6^Li^+^, ^7^Li^+^) and ^55^Mn^+^ are presented. Exemplary active material particles with no delithiation (1, 2) and partial delithiation (3) are highlighted in (b).

The application of a Gaussian function to the histogram data reveals that the signal distribution with the higher intensity ratios of the electrochemically delithiated CAM has a similar mean as the histogram of the pristine NMC532 (see Table S1). This observation therefore indicates the presence of electrochemically inactive NMC532 particles in the electrode, while the lower signal distribution is attributed to delithiated particles. During delithiation, the intensity ratio of Li and Mn decreases since only Li is extracted and the Mn content remains the same (see Table S2), which explains that the lower signal distribution can be assigned to delithiated particles. Furthermore, the diverging intensity ratios account for 15±3 % (sum of the relative number of signals) of the total measured NMC532 particles as obtained by CL‐SP‐ICP‐OES with replicate measurements. These results highlight the importance for a detailed investigation of the local electrochemical environment in the composite electrode since the occurrence of electrochemically inactive particles suggest their disconnection from the electrical network. In Figure 2, the ToF‐SIMS mappings of the cross‐sectional Li and Mn distribution of pristine and electrochemically delithiated NMC532 electrodes are given.

The cross‐section of the pristine NMC532 electrode exhibits a uniform Li distribution indicating a homogeneous mesoscale SOC distribution. The lower apparent intensity in the bottom part of the mapping may be attributable to artifacts of the focused ion beam (FIB) milling (e. g., curtaining). Furthermore, it can be observed that Mn is homogeneously distributed throughout the single‐crystalline CAM particles demonstrating that the concentration is size‐independent, which is further confirmed by EDX (see Figure S2). On the other hand, the cross‐sectional Li distribution in the ToF‐SIMS mapping of the composite electrode after electrochemical delithiation is heterogeneous, which corroborates the non‐uniform Li extraction from the active material as examined by CL‐SP‐ICP‐OES. In Figure S3, selected regions of interest (ROIs) of lithiated and delithiated (ROI 1 and ROI2, respectively) CAM particles of the electrochemically delithiated (SOC of 80±1 %) and of the pristine (ROI 3) NMC532 electrode are presented. The similar intensity of Li in ROI2 and ROI3 corroborates the presence of trapped Li in electrochemically inactive particles. The heterogeneity of the Mn distribution in the ToF‐SIMS mapping after electrochemical delithiation can be attributed to matrix effects.

**Figure 3:**
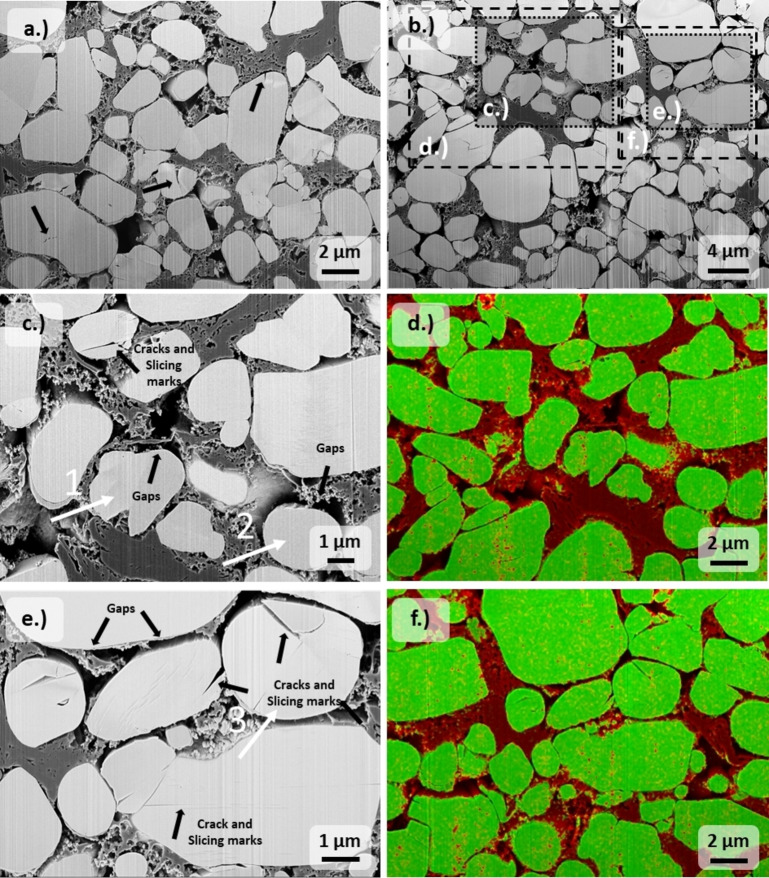
SEM images of FIB‐prepared cross‐sections of (a) pristine and (b) electrochemically delithiated NMC532 (SOC of 80±1 %) electrodes from NMC532‖graphite cells. Furthermore, magnified sections of the electrochemically delithiated NMC532 electrode in (c) and (e) with corresponding EDX elemental mappings in (d) and (f), respectively. For the EDX, an overlay of carbon (red) and oxygen (green) for better visualization of the conductive agent is presented. Exemplary active material particles with no delithiation (1, 2) and partial delithiation (3) are highlighted in (c) and (e) as investigated by ToF‐SIMS.

In SIMS analysis, Li is one of the most sensitive elements due to the low mass and low binding energy leading to a high sputtering yield and thereof preferential ejection from the surface. Therefore, the change of the chemical environment by decreasing the Li content with electrochemical delithiation potentially facilitates the increase of the obtained Mn intensity and explains the heterogeneity of the Mn mapping in the obtained ToF‐SIMS spectrum.[^40^, ^41^, ^42^] For the evaluation of the origins of the observed mesoscale SOC heterogeneity, the microstructure of the composite electrode after delithiation is examined complementarily by means of SEM and EDX (see Figure 3).

The NMC532 particles are embedded in a dense matrix of conductive agent and binder. Unlike typical composite electrodes with polycrystalline CAM, graphite flakes and carbon black particles are usually added to the single‐crystalline active material for increasing the electronic conductivity.[^25^, ^43^] The lithium transport and the electronic resistivity in layer‐structured lithium transition metal oxides is anisotropic.^[44]^ The in‐plane electronic resistivity perpendicular to the *c*‐axis is significantly lower compared to the off‐plane resistivity in parallel direction to the *c*‐axis, which leads to a preferential in‐plane (two‐dimensional) electron transport.^[44]^ The graphite adjuvant constitutes a low‐resistance backbone with long electron transport pathways and a large surface area, which potentially facilitates the electronic integration of the in‐plane facets of the single‐crystals into the conductive network.^[25]^ Polycrystalline CAMs are composited only with nano‐particulate conductive carbon for the improvement of the electronic conductivity of the composite electrode. This results from the random orientation of the primary particles in the secondary particle architecture enabling facile three‐dimensional lithium and electron transport potentially rendering the addition of further graphite additives dispensable.[^14^, ^44^] Furthermore, the high off‐plane resistivity potentially compromises the direct electronic contact between adjacent CAM particles by imposing a dependence on their orientation to each other, which is conceivably more pronounced for single crystals. The EDX elemental mapping of the conductive carbon matrix for NMC532 is further given to highlight the electronic pathways. It can be perceived that the conductive matrix is not homogeneously distributed, which could manifest itself in tortuous electron transport paths and potential isolation from the electronic network. Figure 3c highlights two exemplary NMC532 particles without delithiation as evaluated by ToF‐SIMS. In particular, the second highlighted particle shows a very low degree of surface contact to the conductive carbon matrix. The particle exhibits almost no contact at the left hemisphere, which may not be sufficient for participation in the redox process. On the other hand, the first marked single crystal suggests a higher carbon coverage at the periphery by a rather diffuse carbon matrix. However, the investigated cross‐section does not provide information about the three‐dimensional contact points in the conductive carbon matrix. The percolation of the electrolyte in the pores of the composite electrode enables the ionic transport and hence the transport of Li from/into the CAM particles. Therefore, an insufficient wetting of the NMC532 particles could potentially explain the observed electrochemical inactivity. However, a deficient wetting of individual particles is an improbable explanation for the absent electrochemical redox reactions because in this case inactive domains consisting of several particles would be expected, which is not observed here.

The evolution of micro‐cracks during the calandering process can induce transport hindrances for the electrons.[^16^, ^19^, ^45^, ^46^, ^47^] The single‐crystalline architecture of the active particles has the advantage of high structural consistency and compacted density, which allows the microstructure to withstand high pressure.[^2^, ^48^] These properties lead to the very low extent of microscopic cracks or morphological defects in the pristine NMC532 electrodes, particularly for small particles contrary to polycrystalline NMC532, which possibly excludes this hypothesis as an explanation for the redox inactivity. However, structural inhomogeneity (e. g., cracks) is partially observed in Figure 3a rendering the particle architecture susceptible to stress development upon volume changes during cycling. After electrochemical delithiation at low rates, the formation of further cracks can be observed. The fracture pattern is dominated by crevices on the particle surfaces that penetrate aligned with parallel slicing marks into the interior of the particles, which can be explained by gliding of the layer planes (see Figure 3c,e). The gliding of the layer planes is a mechanism for stress relief as a possible consequence of the inhomogeneous structural stress caused by the development of lithium gradients within the particle.[^13^, ^49^, ^50^, ^51^] These slicing marks were not found in the pristine NMC532 electrode (see Figure 3a). The third highlighted particle shows such a characteristic micro‐crack formation with a deep crevice leading inside. The ToF‐SIMS analysis (third marked particle in Figure 2b) further reveals that the structural compartment on the right side of the observed crack exhibits a higher degree of lithiation, which indicates the persistent confinement of the Li reservoir potentially by electronical contact loss.

The charge of NMC‐based positive electrode materials to high potentials is further accompanied by a significant decrease of the unit cell volume, which leads to the microstructural contraction of the active material.[^52^, ^53^] The volume contraction during delithiation manifests itself in the emergence of irregularly distributed gaps up to 300 nm wide between the active material particles and the carbon matrix (see Figure 3e). This volume change is not buffered by the binder and carbon matrix and could therefore lead to electronic disconnection upon consequent lithiation of the active material during discharge. Furthermore, it is also observed that the carbon matrix is partially ruptured, which prevents its arrangement between the active material particles upon the volume expansion during lithiation.

**Figure 4:**
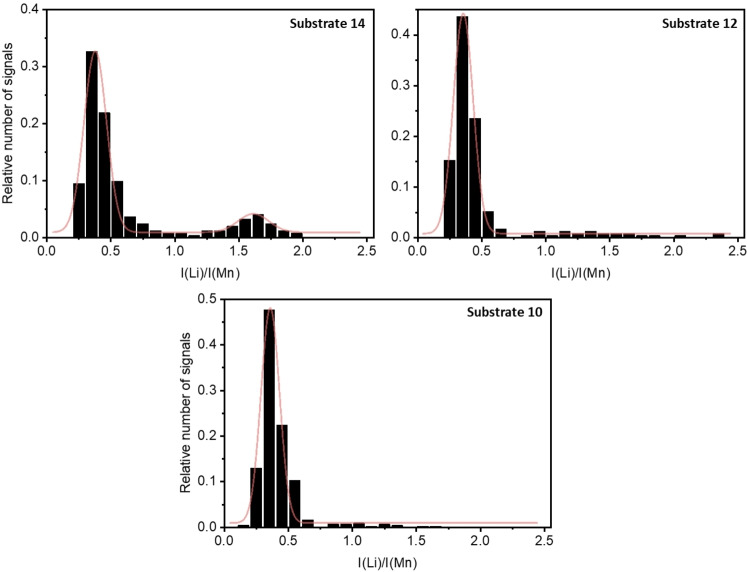
Relative histograms of the NMC532 particle intensity ratios of the different size fractions of electrochemically delithiated NMC532 (SOC of 80±1 %) in NMC532‖graphite cells. The red line indicates the Gaussian fit.

The particle size influence on the homogeneity of the active material utilization is further investigated. For this, particle size fractionation was performed offline by means of low‐pressure cascade impaction before the CL‐SP‐ICP‐OES investigation. The histograms of the collected NMC532 particle sizes from different size fractions are presented in Figure S5. The mode as the most frequent size in the obtained particle size distributions of the different fractions exhibits sizes of 1.7, 1.3, and 0.9 μm for substrate 14, 12, and 10, respectively. An exemplary SEM image of a collected NMC532 particle ensemble from substrate 10 is given in Figure S6. On closer inspection, no sharp fracture edges can be found. This indicates that the incidence of smaller particles is not a result of mechanical fracture, but the result of native occurrence in the particle size distribution. The percentages of the diverging intensity ratios of electrochemically delithiated NMC532 particles of the different size fractions as obtained by low‐pressure cascade impaction are presented in Table 1 and the respective relative histograms are given in Figure 4.

**Table 1:** Percentages of diverging NMC532 particle intensity ratios of the different size fractions of electrochemically delithiated NMC532 (SOC of 80±1 %) in NMC532‖graphite cells.

Substrate	Diverging intensity ratio [%]
14	26±2
12	12±1
10	6±2

The determined percentages of the diverging intensity ratios and hence the mesoscale SOC heterogeneity exhibit a significant dependency on the particle size. The size fractions from the upper collection substrates (e. g., substrate 14) are characterized by an increased mesoscale SOC heterogeneity. This result suggests that smaller particle size as a material property facilitates more uniform delithiation of the single‐crystalline CAM. Furthermore, the active material particles with partial and no electrochemical activity in the ToF‐SIMS mappings are in the size range from 1.6 to 3.3 μm, which confirms this observation. The determined diverging intensity ratios can be attributed to either still fully lithiated or partially delithiated NMC532 particles as shown by ToF‐SIMS.

In Figure 4, the relative histograms of the NMC532 particle intensity ratios after electrochemical delithiation are given. It can be observed that in the case of the size fraction from substrate 14, the distribution of the intensity ratios suggests a combination of non‐ and partially delithiated particles. For the size fractions from substrates 12 and 10, the results suggest the presence of predominantly partially inactive NMC532 particles due to the high relative number of irregularly distributed intensity ratios. As shown in Figure 4, the occurrence of completely non‐delithiated NMC532 particles suggests electrochemical inactivity during the redox reactions, which can be explained by the isolation from the conductive backbone of the composite electrode as discussed above.

The particle size correlates with the length of the Li transport pathways. Although the lithium transport in the active material is the kinetic bottleneck, it is unlikely that solely shorter transport paths for the transient Li movement are a possible explanation for the increased SOC homogeneity after electrochemical delithiation.^[21]^ For the observed particles with only partial delithiation, significant changes (e. g., cracks) preventing the Li transport during the redox reactions are required. As suggested by the particle marked with “3” in Figure 3, the evolution of micro‐cracks upon Li extraction introduces such transport obstacles. The origins of micro‐cracks are diverse as elaborated above. A possible hypothesis for the more uniform delithiation of small particles could be that the cracking mechanism as induced by the gliding of the layer planes due to potential Li concentration gradients is mitigated. Shorter transport ways and thus more facile Li transport could therefore circumvent the buildup of concentration gradients and thus the evolution of micro‐cracks. Furthermore, the increased occurrence of non‐delithiated particles for the largest evaluated size fraction hints at a poorer integration into the conductive matrix.

### Investigation of the mesoscale SOC heterogeneity between different particles upon cycling

The results of the SOC distribution and the microstructural and chemical implications upon cycling are elaborated hereafter. The design of the experiment for the investigation of the mesoscale SOC distribution of NMC532 particles after charge/discharge cycling is identical to our recent publication.^[42]^ Therefore, we refer to the detailed experimental design reported in this reference and will give here only the most important information. The NMC532‖graphite cells were cycled at 1, 3, and 4 C in the voltage range of 3.0 to 4.2 V for 250 cycles (see Figure S7). After charge/discharge cycling, the cells are charged to a cutoff voltage of 4.4 V for the extraction of the active Li from the CAM, which is analogous to the above discussed matrix‐matched external calibration (see Figure 1). Therefore, the determined intensity ratios of the NMC532 particles after cycling are expected to be similarly distributed in the as frames depicted signal distributions corresponding to a mean DOL of 32±<1 %. The percentages of the diverging intensity ratios and the respective relative histograms of the different size fractions of NMC532 particles after charge/discharge cycling at different rates are given in Table 2 and Figures S8–S10, respectively. The SOC heterogeneity increases particularly for the size fraction of substrate 14 after cycling, which implies the evolution of structural (e. g., cracks) or chemical changes upon repeated (de)lithiation. Furthermore, it can be observed that the SOC distribution is more uniform after the application of higher C‐rates (e. g., 3 or 4 C) compared to a moderate C‐rate of 1 C.

**Table 2:** Percentages of diverging NMC532 particle intensity ratios of the different size fractions after electrochemical cycling in NMC532‖graphite cells under different cycling protocols at 3.0–4.2 V for 250 cycles and a final charging step to a cutoff voltage of 4.4 V.

Substrate	Diverging intensity ratio [%]		
	1 C	3 C	4 C
14	39±2	34±2	29±3
12	14±2	11±2	14±3
10	6±1	5±2	6±1

**Figure 5:**
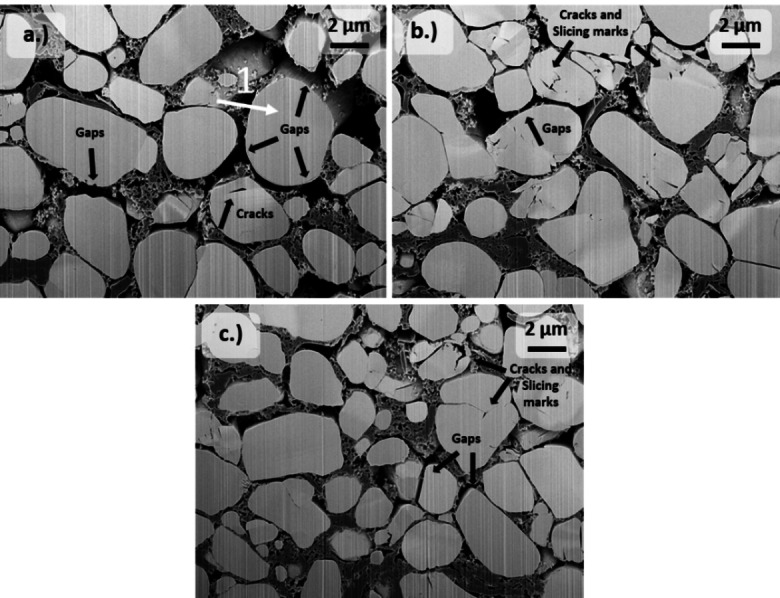
SEM images of FIB‐prepared cross‐sections of NMC532 electrodes in discharged state after charge/discharge cycling in NMC532‖graphite cells under different cycling protocols at 3.0–4.2 V with (a) 1 C, (b) 3 C, and (c) 4 C for 250 cycles, respectively.

Therefore, the microstructure of the composite electrode after cycling is examined comprehensively by SEM (see Figure 5). The application of a moderate cycling rate of 1 C leads to the distributed formation of wide gaps between the active material particles and the conductive matrix (e. g., >400 nm for the first highlighted particle in Figure 5). Since the SEM investigation of the electrode cross‐section is performed in discharged state after cycling, this observation suggests the structural fatigue of the composite matrix (e. g., binder, carbon network). The repeated anisotropic volume change of the active material during cycling is obviously not absorbed within the composite framework, which is reflected in its partial deformation. This structural fatigue of the composite matrix could therefore explain the significant increase in SOC heterogeneity after cycling at 1 C, as the (de)lithiation of the CAM is hindered.

The evolution of gaps between the active material particles is also observed after charge/discharge cycling at 3 and 4 C, but to a lesser extent. As mentioned above, the Li (de)insertion during cycling correlates to a repeated change of the unit cell volume of the active material, which is dependent on the amount of extracted Li.[^52^, ^53^] The application of higher C‐rates leads to less quantitative (de)lithiation reactions in the respective potential range due to kinetic limitations resulting in lower achieved capacities (see Figure S7). Therefore, the corresponding volume change of the active material is less pronounced, which potentially alleviates the structural stress on the composite matrix and hence leads to a longer‐lasting conductive network. For the size fractions of substrate 12 and 10, no increase of the SOC heterogeneity after cycling is observed considering the standard deviation. The lower absolute volume change during (de)lithiation of smaller particles assumedly helps to preserve the surrounding composite structure. The formation of microscopic cracks within the NMC532 particles is more distinctive after cycling with higher current densities. The development of Li concentration gradients at high C‐rates can lead to spatially heterogeneous lattice volume changes and is therefore a potential driving force for the observed formation of slicing marks and cracks in single‐crystals.[^8^, ^13^] However, considering the decreased SOC heterogeneity with higher C‐rates and the low extent of particle cracking after cycling at 1 C, CAM particle cracking is not the dominant cause for non‐uniform active material utilization. The emergence of cracks can enable the penetration of the electrolyte into the particle interior leading to new electrochemically active surface areas, which potentially bypass evolved Li transport barriers such as electrical resistant surface films (e. g., rock‐salt structures).[^6^, ^54^] However, the developed slicing marks and cracks rarely propagate completely inside the particle, which excludes this as a possible explanation for the lower SOC heterogeneity with high C‐rates. But it has to be noted that cracks in the nanometer regime, which are hardly resolved by SEM, can potentially lead to electrolyte infiltration in the interior.^[54]^ The formation of inactive phases at the cathode|electrolyte interface of the NMC532 particles is a further conceivable reason for the observed occurrence of persistent SOC heterogeneity. The surface reconstruction (e. g., buildup of rock‐salt phases) of the CAM particles occurs upon prolonged Li deficiencies and leads to the potential inhibition of the Li transport by increasing the interfacial resistance.[^9^, ^13^, ^27^, ^49^, ^55^] Although the application of high current densities facilitates kinetic differences of the Li transport between the particle surface and interior entailing non‐uniform (de)lithiation of the CAM, more homogeneous active material utilization results for higher C‐rates. Furthermore, the formation of a thick and non‐uniform CEI upon spontaneous chemical or electrochemical degradation during cycling can further affect the Li (de)insertion.[^28^, ^29^, ^30^, ^31^] The significantly more uniform SOC distribution for smaller active particle sizes after cycling, however, further questions the impact of surface‐dependent failure mechanisms for the evolution of SOC heterogeneity. Small particles have a larger ratio of the surface area to the volume, and therefore the influence of such interfacial side reactions is increased. Therefore, the shorter transport pathways in the particle bulk could also prevent the evolution of compositional gradients of the Li distribution and as mentioned above, these compositional gradients promote, among other things, the surface reconstruction. The occurrence of transition metal dissolution (TMD) as a surface‐dependent aging phenomenon was also investigated by means of ICP‐OES in solution mode and considered negligible due to the very low amount of deposited transition metals on the anode after cycling (see Table S2). Therefore, it is concluded that surface degradation phenomena or particle cracking are not the dominant reasons for the observed SOC heterogeneity and the conductive network provided by the composite electrode microstructure and its durability during cycling plays a more important role for the non‐uniform capacity utilization.

## Conclusion

The particle size‐dependent evolution of persistent mesoscale state‐of‐charge (SOC) heterogeneity was observed after slow delithiation and extensive relaxation times with classification‐single‐particle inductively coupled plasma optical emission spectroscopy (CL‐SP‐ICP‐OES). The investigation by means of time of‐flight secondary ion mass spectrometry (ToF‐SIMS) revealed the presence of inactive Li reservoirs in non‐ and partially delithiated cathode active material (CAM) particles. The investigation of the composite electrode via scanning electron microscopy (SEM) and energy‐dispersive X‐ray spectroscopy (EDX) suggested the isolation from the electrical network for the non‐delithiated particles due to the insufficient distribution of the conductive matrix. Furthermore, the SOC distribution between different CAM particles was observed to be size‐dependent with the largest evaluated size fraction exhibiting the highest SOC heterogeneity. For the partially delithiated particles, crevices on the particle surfaces that penetrate aligned with parallel slicing marks into the interior of the particles were observed. ToF‐SIMS analysis revealed the persistent confinement of the Li reservoir potentially by electronic contact loss. After charge/discharge cycling, an increase of the SOC heterogeneity was found for the largest size fraction. Furthermore, the development of wide gaps between the CAM particles was observed in discharged state and concluded to originate from the structural fatigue of the composite matrix (e. g., binder, carbon network). The extent of the structural fatigue was dependent on the current density (1 C>3 C>4 C). This observation correlates with the decreased SOC heterogeneity at higher cycling rates. The formation of microscopic cracks within the CAM particles is more distinctive after cycling with higher current densities. However, considering the decreased SOC heterogeneity with higher C‐rates and the low extent of particle cracking after cycling at 1 C, CAM particle cracking is not the dominant cause for the SOC heterogeneity. Furthermore, the significantly more uniform CAM utilization of the smallest evaluated size fractions particularly at high rates questions the impact of surface‐dependent failure mechanisms for the evolution of SOC heterogeneity. Therefore, it is concluded that for the investigated single‐crystalline CAM, a deficient structural flexibility of the composite electrode framework is the main driver for the observed non‐uniform CAM utilization.

## Experimental Section

### Electrochemical cycling

In this study, commercially available NMC532‖graphite pouch cells (two‐electrode configuration) were used from Li‐Fun Technology (China) with geometric dimensions of 35×20×4 mm.^[56]^ This cell setup was chosen because of the high absolute active material content (≈1.1 g), which is needed for the size fractionation by means of low‐pressure cascade impaction. The cell chemistry was based on single‐crystalline NMC532 (94.0 wt %) with polyvinylidene difluoride (2.0 wt %) and carbon black (4.0 wt %). The negative electrode was based on artificial graphite (94.8 wt %), styrene‐butadiene‐rubber (2.5 wt %), sodium carboxymethyl cellulose (1.3 wt %), and carbon black (1.4 wt %). For the assembly, the NMC532‖graphite pouch cells were filled with 700 μL of 1 m LiPF_6_ in ethylene carbonate/ethyl methyl carbonate (EC/EMC, 30 : 70 wt %) and sealed (165 °C, 90 kPa, 5 s) using a vacuum sealer from Gelon LIB Group (China) in a dry room. The prefabricated cells were dried for 24 h at 60 °C under reduced pressure (0.001 mbar) before electrolyte filling in a dry room (dew point −65 °C). For the positive and negative electrode, the mass loading was around 13.7 and 10 mg cm^−2^ as given by the manufacturer, respectively. The separator used was based on polyethylene (PE) with one‐sided Al_2_O_3_‐coating towards the positive electrode. For controlled pressure in the cell, the sealed pouch cells were clamped into a specifically designed cell holder.^[57]^

The electrochemical procedure of the matrix‐matched external calibration was composed of a constant current–constant voltage (CCCV) charging step. The NMC532‖graphite cells were charged to SOCs of 22±<1 %, 41±<1 %, 60±<1 %, and 80±1 % with charge cut‐off voltages of 3.6, 3.7, 4.0, and 4.4 V at a specific current of 6 mA g^−1^, respectively. The SOC was calculated based on the obtained capacity during the charge process. The theoretical capacity of 278 mAh g^−1^ was used for this, since this value represents the absolute Li content. The cut‐off voltages have been determined in time‐limited CC charge cycling experiments with specified practical capacities of 56, 111, 167 m and 222 mAh g^−1^, respectively.

The electrochemical procedure of the formation and the subsequent cycling was performed with CCCV charging and CC discharging. The formation for the cycling experiments consisted of two formation cycles in the voltage range of 3.0 to 4.2 V at 0.1 and 0.2 C. The NMC532‖graphite cells were cycled at 1, 3, and 4 C in the voltage range of 3.0 to 4.2 V. After this cycling procedure, the cells were charged to a cut‐off voltage of 4.4 V at 6 mA g^−1^, enabling the extraction of the mobile Li from the cathode active material for CL‐SP‐ICP‐OES. For the cycling experiments, the specific current at 1 C was defined as 160 mA g^−1^.

### ICP‐OES investigations

The CL‐SP‐ICP‐OES and ICP‐OES measurements were performed with an ARCOS from SPECTRO Analytical Instruments GmbH (Germany). The emission lines of Li (670.776 nm) and Mn (257.611 nm) were acquired with axial plasma viewing and Mn was selected for analysis because of the higher intensity of the emission lines compared to Ni and Co. Before analysis, the pouch cells were disassembled in a dry room and the NMC532 electrodes were rinsed with 1 mL EMC. For the investigation of the mesoscale SOC distribution with CL‐SP‐ICP‐OES, the CAM particles need to be removed from the composite electrode. Therefore, ultrasonic treatment with the UP100H ultrasonic processor from Hielscher Ultrasonics GmBH (Germany) in *N*‐methyl‐2‐pyrrolidone (NMP) under elevated temperature was performed for the delamination of the electrode. Afterwards, the formed suspension was centrifuged, and the particulate residue was dried under reduced pressure. For the evaluation of the electrochemical delithiation for the matrix‐matched external calibration, the electrodes were examined by acidic microwave digestion and subsequent ICP‐OES analysis in solution mode described by Vortmann‐Westhoven et al.^[58]^ The negative electrodes were further studied analogously to investigate the possible occurrence of TMD after cycling.

### SEM, EDX, and TOF‐SIMS investigations

SEM with a field emission gun (Schottky‐type) was performed to investigate the surface of the electrodes and the particle size and architecture. The measurements were carried out on multiple areas of the sample using an Auriga CrossBeam workstation from Zeiss (Germany). Cross‐sections were prepared by means of FIB milling using gallium ions generated from a liquid metal ion source. EDX was conducted to examine the elemental composition of the surface and cross‐section on multiple areas of the sample. The measurements were performed with an accelerating voltage of 15 kV using an Ultim® Extreme EDX detector and evaluated with the INCA software, both from Oxford Instruments (United Kingdom). ToF‐SIMS was performed using a TOF.SIMS 5 instrument from ION TOF GmbH (Germany). The nanoscale elemental mapping of the cross‐section was carried out with a liquid metal bismuth ion source (Bi^+^ 30 keV) in the imaging mode combined with delayed secondary ion extraction. The positive electrodes were analyzed by SEM, EDX, and ToF‐SIMS after washing with 1 mL EMC.

### Low‐pressure cascade impaction

Low‐pressure cascade impaction (LPI) was performed using the Dekati® ELPI®+ from Dekati Ltd. (Finland). Prior to the introduction into the cascade impactor, the particles were injected from above into a custom‐made glass cylinder made of borosilicate glass by the glassblowing workshop of the inorganic chemistry institute of the University of Münster. This procedure was done for the suspension of the particles in the air draft induced by the applied low‐pressure (40 mbar) of the cascade impactor. The particles were injected by means of a 3 mL syringe from Braun (Germany) equipped with a cannula (1.0×120 mm) from neoLab Migge GmbH (Germany). A total mass of around 300 mg was introduced in small batches for each sample. After particle suspension, the generated aerosol was passed through a cyclone from Dekati Ltd. (Finland) for the pre‐cut of large agglomerates. The aerosol sample flow was then directed into the cascade impactor. The connections between the glass cylinder, the cyclone and the cascade impactor were enabled by BEV‐A‐LINE® XX tubings (inner diameter: 9.5 mm, wall thickness: 1.6 mm, outer diameter: 12.7 mm) from OPTUBUS (Germany). The size fractions of impactor stage 14, 12, 10 and the Pre‐LPI (PreLPI) sample were investigated by CL‐SP‐ICP‐OES. The SEM images of the collected particle ensembles after the classifier were evaluated with ImageJ 1.50i (USA) to obtain the particle size distributions.

## Conflict of interest

The authors declare no conflict of interest.

1

## Supporting information

As a service to our authors and readers, this journal provides supporting information supplied by the authors. Such materials are peer reviewed and may be re‐organized for online delivery, but are not copy‐edited or typeset. Technical support issues arising from supporting information (other than missing files) should be addressed to the authors.

Supporting Information

## Data Availability

The data that support the findings of this study are available from the corresponding author upon reasonable request.
