# Supplementary material for: State‐of‐Charge Distribution of Single‐Crystalline NMC532 Cathodes in Lithium‐Ion Batteries: A Critical Look at the Mesoscale
Source: ChemSusChem. 2022 Oct 1;15(21):e202201169. doi: 10.1002/cssc.202201169 (PMC9828165; doi:10.1002/cssc.202201169)
Supplement: Supplementary file 1 — Supporting Information [file CSSC-15-0-s001.pdf]

ChemSusChem

Supporting Information

State-of-Charge Distribution of Single-Crystalline NMC532 Cathodes in Lithium-Ion Batteries: A Critical Look at the Mesoscale

Till-Niklas Kröger, Mathis Jan Wölke, Patrick Harte, Thomas Beuse, Martin Winter, Sascha Nowak, and Simon Wiemers-Meyer*© 2022 The Authors. ChemSusChem published by Wiley-VCH GmbH. This is an open access article under the terms of the Creative Commons Attribution License, which permits use, distribution and reproduction in any medium, provided the original work is properly cited.

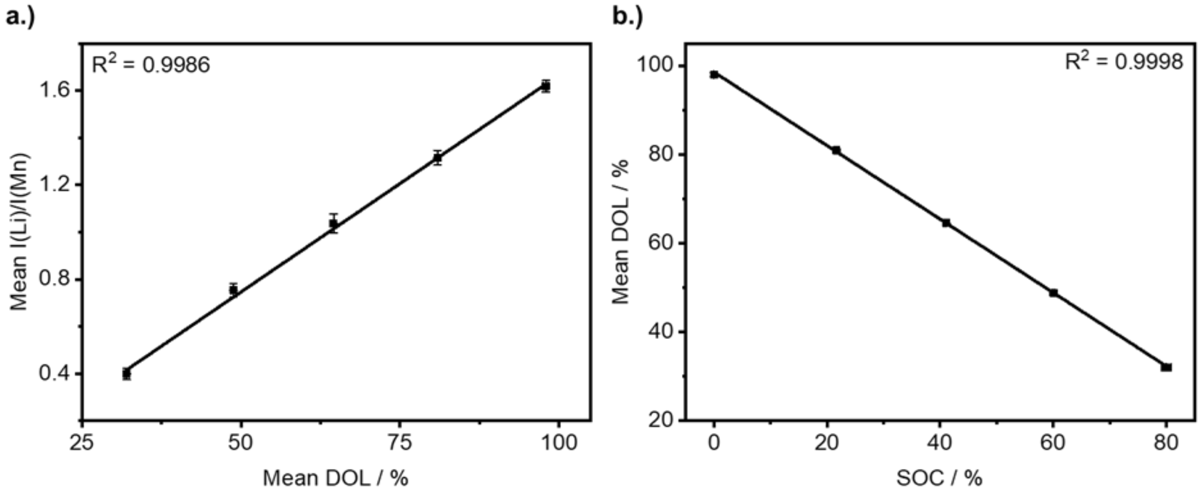

Figure S1: Mean peak intensity ratios in a.) for different mean DOLs and mean DOLs in b.) for different SOCs in NMC532‖graphite cells (two-electrode configuration) as obtained by CL-SP-ICP-OES and after acidic microwave digestion with ICP-OES in solution mode, respectively.[1] The SOC is determined based on the obtained capacity during the charge process.

Table S1: Summary of the mean peak intensity ratios and the relative standard deviations of Gaussian fitted signal distributions for different SOCs of NMC532 particles.

Sample	SOC / %	Mean I/(Li)/I(Mn)	RSD / %	Reproducibility / %
NMC532	0	1.6	8.2	1.8
	80 ± 1 %	0.4	18.7	3.3
		1.6	8.7	1.9

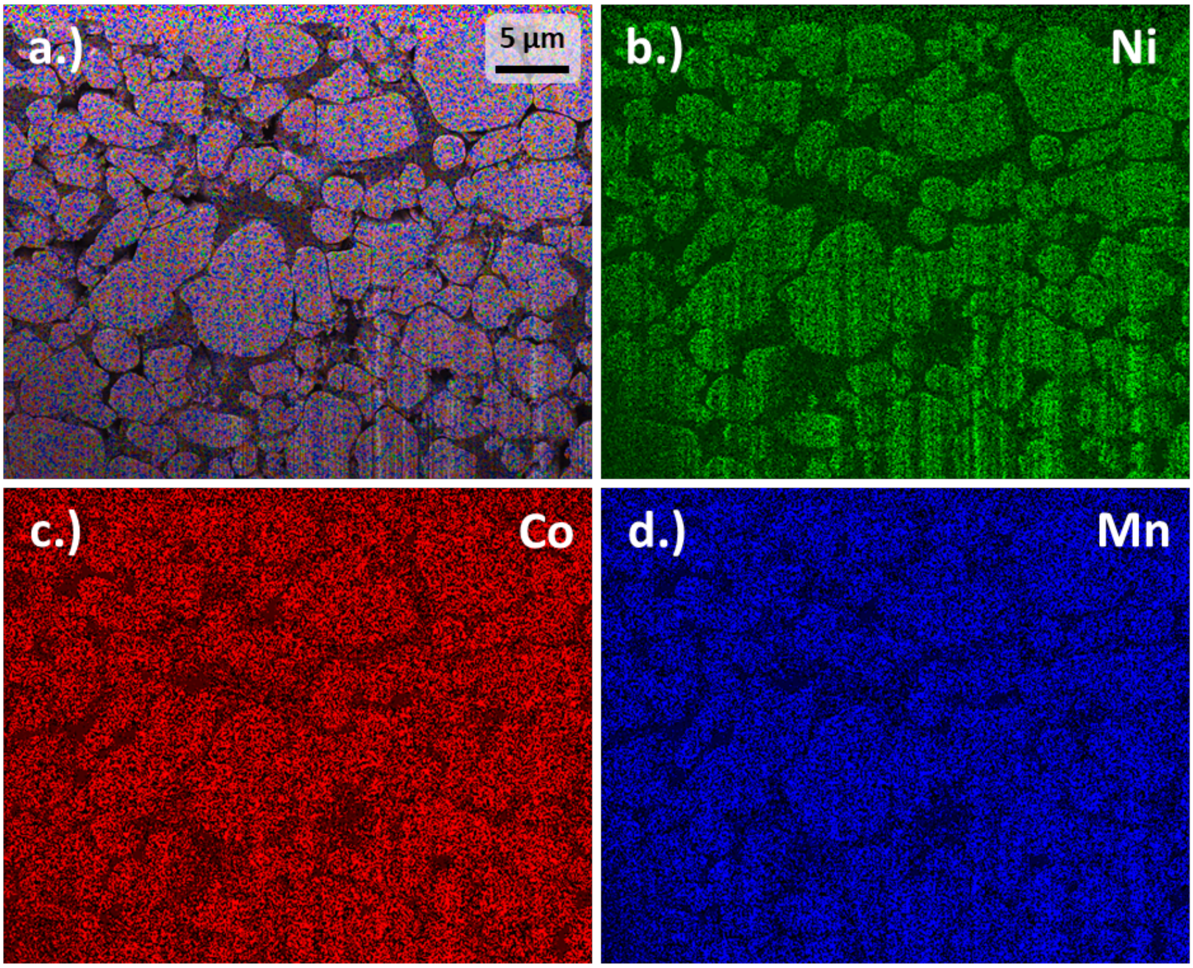

Figure S2: EDX elemental mappings collected on a FIB-prepared cross-section of electrochemically delithiated NMC532 (SOC of 80 ± 1 %) electrode in NMC532ǁgraphite cells with a.) an overlay of the Ni, Co and Mn elemental distribution and b.), c.) and d.) the Ni, Co and Mn elemental distribution, respectively.

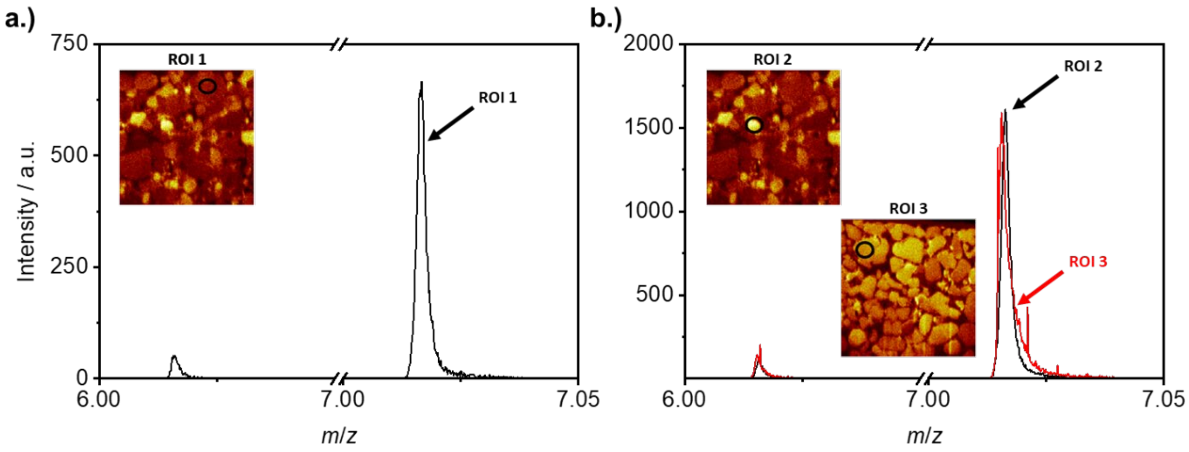

Figure S3: ROI (black circles) analysis of ToF-SIMS spectra ($^{6}Li^{+}$, $^{7}Li^{+}$) collected on an electrochemically delithiated SC-NCM523 (SOC of 80 ± 1 %) electrode with ROI 1 (delihtiated particle) and ROI 2 (lithiated particle) and on a pristine SC-NCM523 electrode with ROI 3 (lithiated particle) from SC-NMC532ǁgraphite cells.

Particle size fractionation by low-pressure cascade impaction

The working principle of the cascade impaction is depicted schematically in **Figure S4**. The cascade impactor consists of multiple impactor stages, each with two co-linear plates, the jet plate and the collection plate. The aerosol sample flow passes first the jet plate and due to the higher inertia, larger particles impact onto the collector plates, where they are collected on aluminum substrates. Smaller particles remain in the sample flow and are impacted onto the collection substrates further down the cascade impactor as the nozzle size of the jet plates decreases.

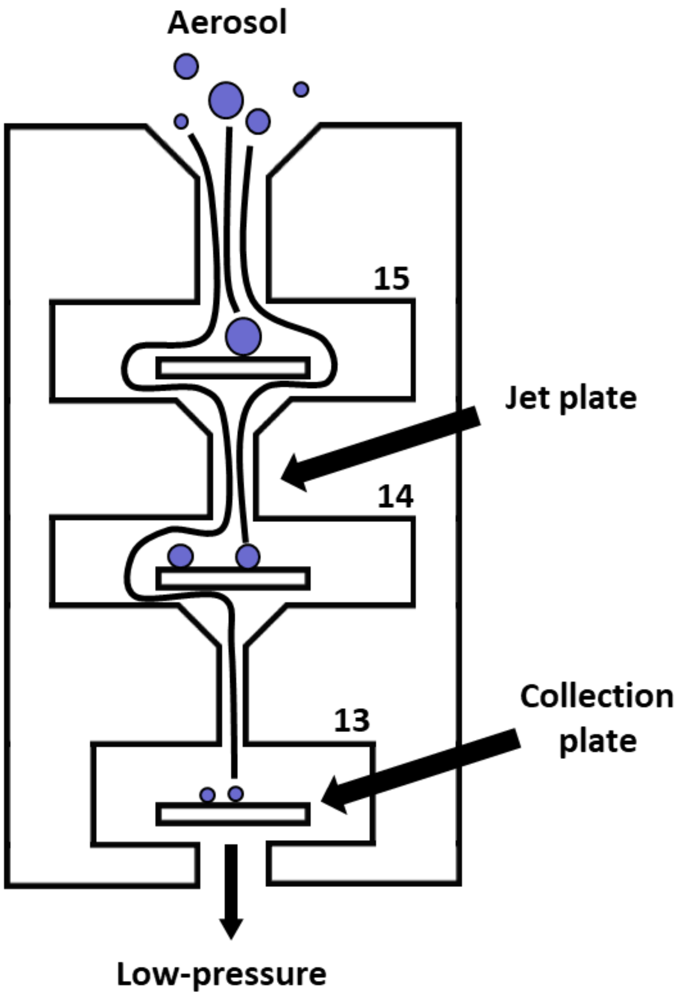

Figure S4: Schematic representation of the working principle of the particle size fractionation *via* low-pressure cascade impaction with exemplary substrate labeling (15, 14, 13). Adapted from.[2]

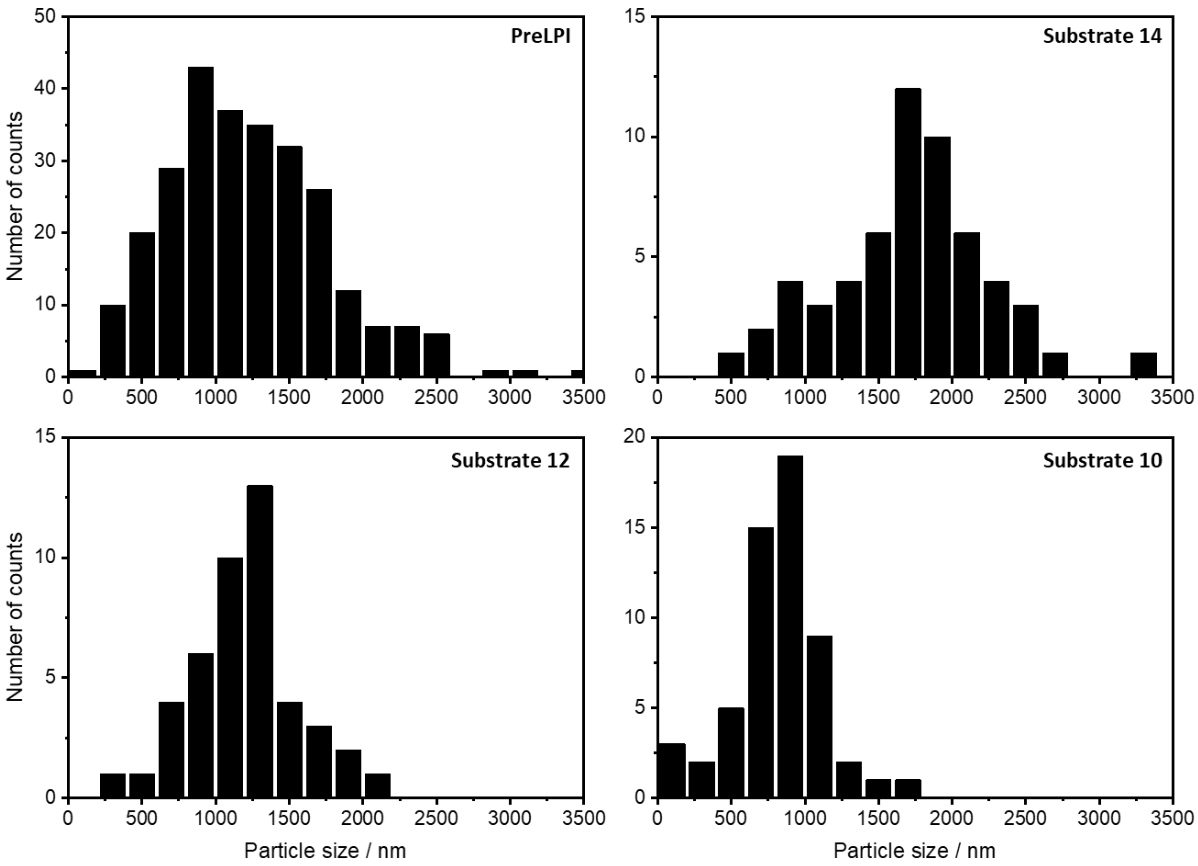

Figure S5: Histograms of the collected NMC532 particle sizes from different size fractions obtained by processing of SEM images. The respective particle ensembles were collected by sampling the particle aerosol on a SEM sample holder before entering the ICP. The size fractionation of substrate 14, 12 and 10 was performed by means of low-pressure cascade impaction.

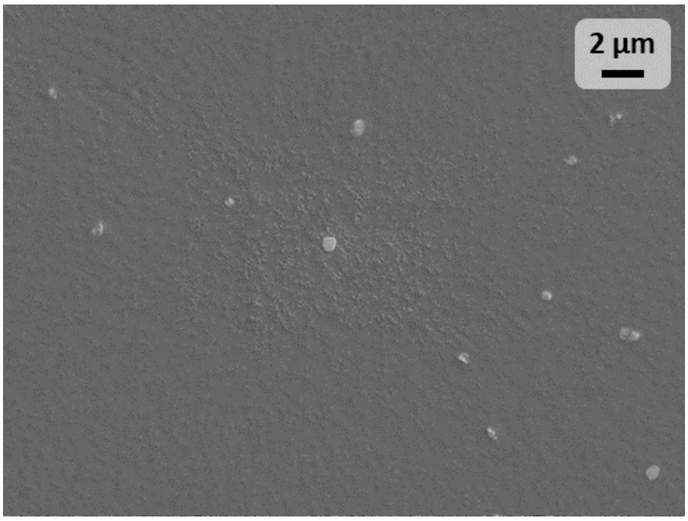

Figure S6: SEM image of a collected NMC532 particle ensemble from substrate 10 after the classifier. The particle ensemble was collected by sampling the particle aerosol on a SEM sample holder before entering the ICP. The size fractionation was performed by means of low-pressure cascade impaction.

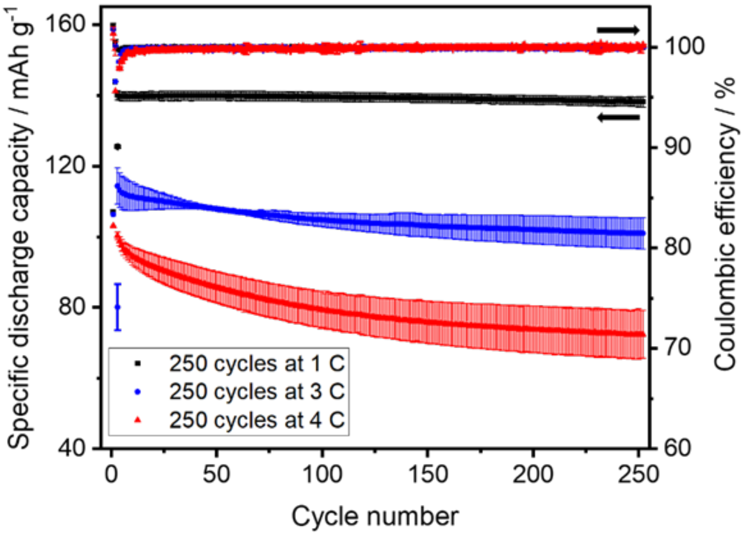

Figure S7: Comparison of the specific discharge capacities and the Coulombic efficiencies of NMC532‖graphite full cells (two electrode configuration) in the cell voltage range of 3.0 to 4.2 V for 250 cycles at 1 C, 3 C and 4 C after two formation cycles at 0.1 C and 0.2 C in the same voltage range.

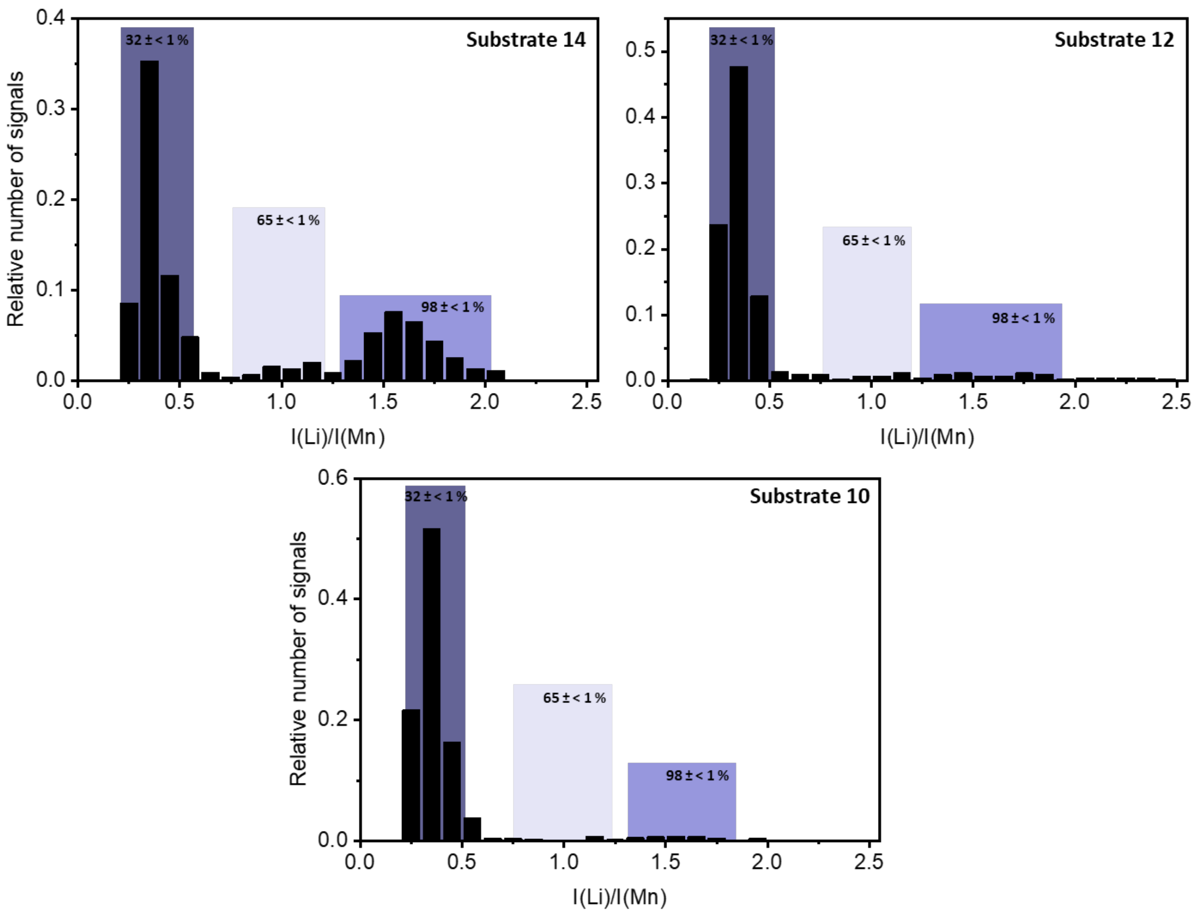

Figure S8: Relative histograms of the NMC532 particle intensity ratios of different size fractions after charge/discharge cycling in NMC532ǁgraphite cells with 1 C for 250 cycles at 3.0 V to 4.2 V and a final charging step to a cutoff voltage of 4.4 V as obtained by CL-SP-ICP-OES. Three replicate measurements were performed and depicted in the same histogram. The colored frames represent the histograms of the matrix-matched external calibration with a width correlating to the ± 2 σ standard deviation of the corresponding mean intensity ratio.

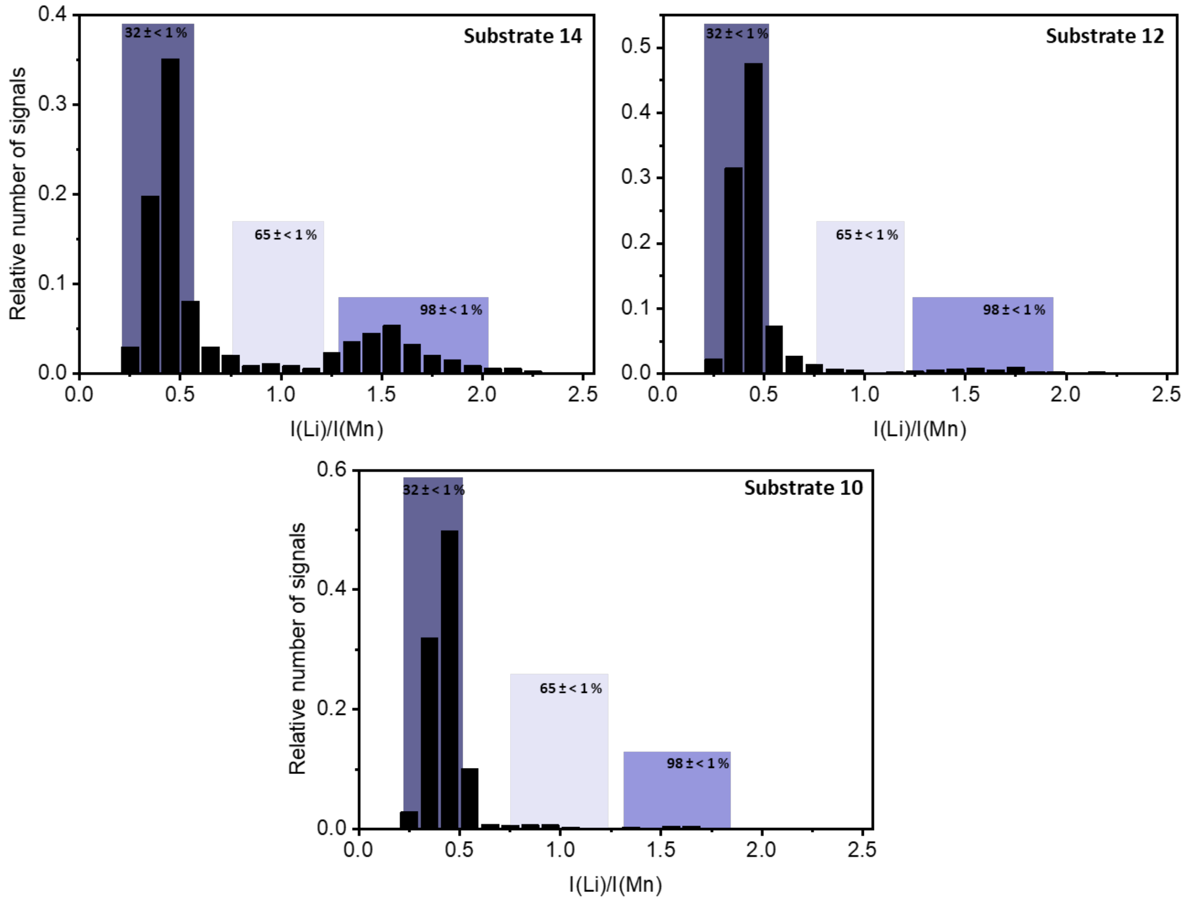

Figure S9: Relative histograms of the NMC532 particle intensity ratios of different size fractions after charge/discharge cycling in NMC532‖graphite cells with 3 C for 250 cycles at 3.0 V to 4.2 V and a final charging step to a cutoff voltage of 4.4 V as obtained by CL-SP-ICP-OES. Three replicate measurements were performed and depicted in the same histogram. The colored frames represent the histograms of the matrix-matched external calibration with a width correlating to the ± 2 σ standard deviation of the corresponding mean intensity ratio.

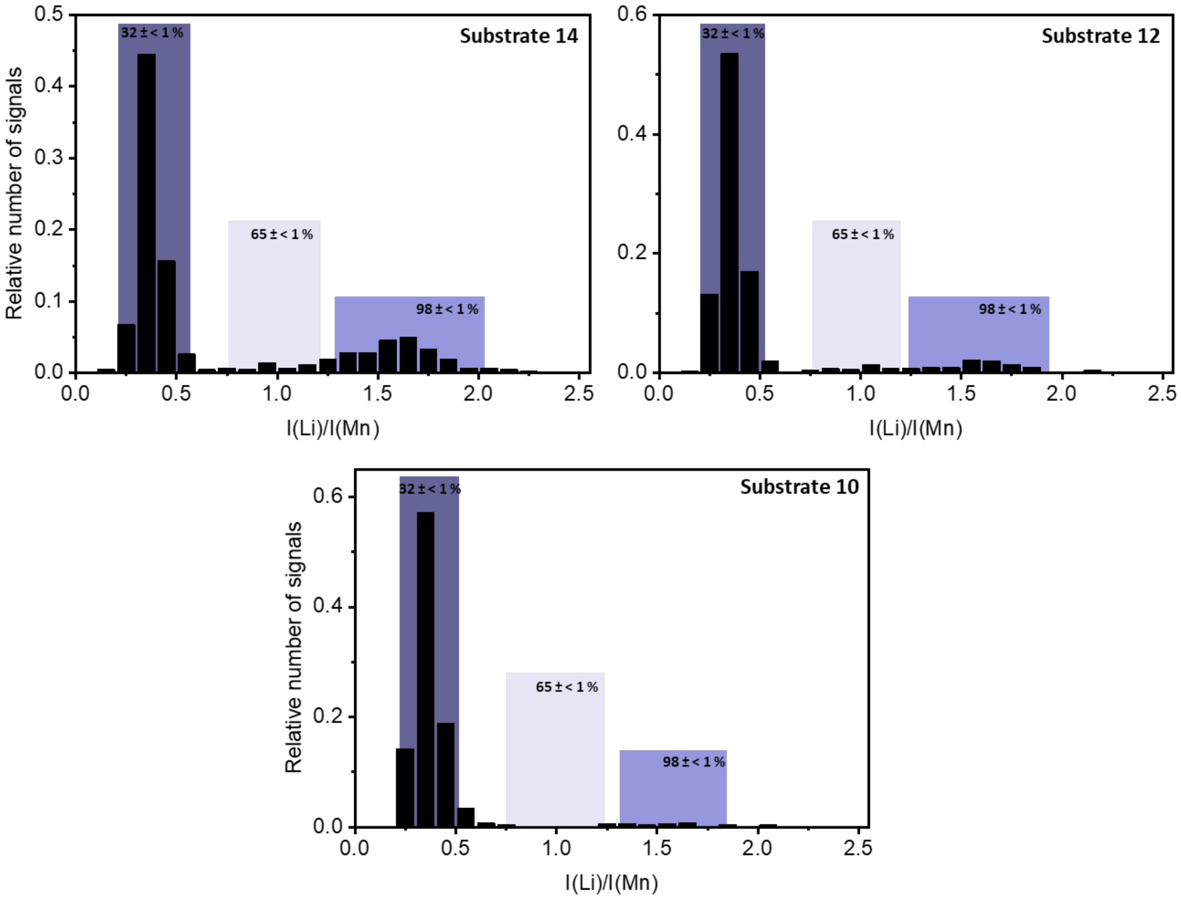

Figure S10: Relative histograms of the NMC532 particle intensity ratios of different size fractions after charge/discharge cycling in NMC532||graphite cells with 4 C for 250 cycles at 3.0 V to 4.2 V and a final charging step to a cutoff voltage of 4.4 V as obtained by CL-SP-ICP-OES. Three replicate measurements were performed and depicted in the same histogram. The colored frames represent the histograms of the matrix-matched external calibration with a width correlating to the ± 2 σ standard deviation of the corresponding mean intensity ratio.

Table S2: Overview of the transition metal deposition on the anode after different cycling procedures in discharged state and after electrochemical delithiation to a cutoff voltage of 4.4 V in NMC532‖graphite cells as obtained with ICP-OES in solution mode. The limits of quantification (LOQ) for the investigated elements are 11 ng cm^{-2}, 65 ng cm^{-2} and 82 ng cm^{-2} for Ni, Co and Mn, respectively.

Procedure	Ni / µg cm^{-2}	Co / µg cm^{-2}	Mn / µg cm^{-2}
250 cycles at 1 C	0.75 ± 0.09	< LOQ	0.14 ± 0.04
250 cycles at 3 C	0.88 ± 0.07	< LOQ	0.22 ± 0.06
250 cycles at 4 C	1.40 ± 0.11	< LOQ	0.26 ± 0.05
Charge to 4.4 V	< LOQ	< LOQ	< LOQ

References

1. R. Nölle, K. Beltrop, F. Holtstiege, J. Kasnatscheew, T. Placke, Winter, M. *Mater. Today* **2020**, *32*, 131–146.

2. Dekati® ELPI® Manual (Version 1.55), Dekati Ltd., **2018**.

Author Contributions

Till-Niklas Kröger (experimental investigation, data interpretation, validation, writing of original draft; lead contribution), Mathis Jan Wölke (data interpretation, experimental investigation; supporting contribution), Patrick Harte (data interpretation, experimental investigation; supporting contribution), Thomas Beuse (experimental investigation; supporting contribution), Sascha Nowak (data interpretation, proofreading; supporting contribution), Martin Winter (data interpretation, proofreading; supporting contribution), Simon Wiemers-Meyer (data interpretation, proofreading; supporting contribution).
